# Shoulder Tendinopathy Induced by Statins: A Case Report and Systematic Review

**DOI:** 10.3390/jpm15050198

**Published:** 2025-05-15

**Authors:** Nicola Manocchio, Carmelo Pirri, Andrea Sorbino, Laura Giordani, Giulia Vita, Concetta Ljoka, Calogero Foti

**Affiliations:** 1 Physical and Rehabilitation Medicine, Clinical Sciences and Translational Medicine Department, Tor Vergata University, 00133 Rome, Italy; andrea.sorbino@ptvonline.it (A.S.); giordani@med.uniroma2.it (L.G.); giulia.vita@students.uniroma2.eu (G.V.); concetta.ljoka@ptvonline.it (C.L.); 2 PhD Course in Tissue Engineering and Remodeling Biotechnologies for Body Function, Tor Vergata University, 00133 Rome, Italy; 3 Department of Neurosciences, Institute of Human Anatomy, University of Padova, 35121 Padua, Italy; carmelo.pirri@unipd.it

**Keywords:** hyaluronic acid, intra-articular injection, mesotherapy, rehabilitation, rotator cuff injury, statins, steroids, tendinopathy, therapeutic exercise (exercise therapy)

## Abstract

**Background:** Statins are essential for managing cholesterol levels but can induce musculoskeletal side effects, including tendinopathy of the shoulder. Rotator Cuff Disease (RCD) is one of the most common shoulder tendinopathy. The aim of the present study is to report a clinical case of statin-induce RCD after performing a systematic review on the subject. **Materials and Methods:** We performed a systematic review of the literature and report the case of a 49-year-old man with statin-induced RCD treated with a personalized individual rehabilitation project (IRP) (steroid and HA injections, mesotherapy, and therapeutic exercise) to investigate the relationship between statins and shoulder tendinopathy. The review followed PRISMA guidelines (2020 version), searching PubMed, Web of Science, and SCOPUS. **Results:** Out of a total of 217 articles, three cohort studies were suitable for our review. Conflicting evidence emerged regarding the association between statins and shoulder tendinopathy from the included papers. The case report describes a patient who experienced RCD after increasing atorvastatin dosage, with symptoms improving after dose reduction and a multimodal personalized IRP. **Conclusions:** Statins may contribute to tendon injury by altering the extracellular matrix and cell membrane integrity. While tendinopathy and statin relation is still under discussion, clinicians should monitor patients for tendinopathy and consider switching to alternative treatments in case symptoms arise. The case report demonstrated the successful management of statin-induced RCD with a multimodal personalized IRP. Further research is needed to clarify the relationship between statins and shoulder tendinopathy. Early diagnosis and appropriate personalized management are crucial for optimizing patient outcomes.

## 1. Introduction

The first statin, lovastatin, was introduced in clinical practice three decades ago. Since then, the statin class has expanded, becoming an essential tool for managing cholesterol levels and preventing cardiovascular disease, both in primary and secondary prevention [1]. Statins act by inhibiting the 3-hydroxy-3-methylglutaryl coenzyme A reductase, the rate-limiting enzyme in cholesterol biosynthesis [2]. Common side effects of statin use include myalgias, myopathy, rhabdomyolysis, and elevated liver enzymes [3]. Atorvastatin, alongside simvastatin and lovastatin, carries a higher risk of side effects due to the nonselective diffusion into tissues beyond the liver [4]. Recent research has highlighted the potential for statins to induce tendinopathy but with conflicting reports. Case reports and pharmacovigilance data suggest an increased risk of these complications in statin users within the first year of treatment, particularly in male patients [5,6]. Tendinopathy is a broad term for tendon inflammation and degeneration. Tendinopathy, particularly of the rotator cuff [i.e., Rotator Cuff Disease (RCD)], is closely associated with shoulder function impairments, pain, and exercise intolerance; beyond the affected shoulder, RCD can also impact the contralateral side [7]. Hyaluronic acid (HA), a natural polysaccharide found in synovial fluid, is a promising treatment option for RCD due to its viscoelastic, lubricating, and anti-inflammatory qualities [8,9,10,11].

In situations as complex as statin-induced RCD, physiatrists may intervene with a multimodal personalized individual rehabilitation project (IRP) [12]. IRPs aim to restore independence, enhance social participation, and improve the quality of life by following a structured process: comprehensive assessments, goal setting based on patient needs, and the selection of outcome measures to track progress. Tailored interventions (e.g., motor re-education, injection therapy, and education) are delivered by specialized professionals. IRPs are continuously monitored and adjusted to optimize outcomes [13].

The aim of the present study is to report a clinical case of statin-induced shoulder tendinopathy to highlight statin and shoulder tendinopathy relation and consequent management through a personalized IRP. Additionally, a systematic review was conducted to evaluate the existing literature on the effects of statins on shoulder disease.

## 2. Materials and Methods

A systematic review of the literature was conducted according to the PRISMA guidelines (2020 version) [14]. The following algorithm was established:

• P (Problem): shoulder tendinopathy
• I (Intervention): statin treatment
• C (Comparison): statin-induced shoulder tendinopathy
• O (Outcomes): Rate of occurrence of shoulder tendinopathy during statin treatment

A systematic search was performed in the online databases of PubMed, Web of Science, and SCOPUS from the start of databases coverage to the 10th of October 2024. The protocol for this systematic review was duly registered with Open Science Framework registries accessible via the following registration link: https://doi.org/10.17605/OSF.IO/DSHFY. The following MESH (Medical Subject Headings) terms with the relative Boolean operators were used for Database search: “(Statin OR Statins OR Hydroxymethylglutaryl CoA Reductase Inhibitors) AND (Shoulder OR Tendinopathy OR Shoulder Pain OR Shoulder Injuries OR Rotator Cuff Injuries OR Shoulder Impingement Syndrome OR Tendon Injuries)”. No filter (time or others) was set for the search.

Two authors (NM and GV) performed the database search. In the case of disagreements, a third author (CP) with long experience on systematic reviews settled the matter. Duplicates were checked and deleted after manual revision. Clinical trials (randomized or not), retrospective studies, and cohort studies which involved statin-induced shoulder-injury were considered for inclusion only if they were in English. Reviews, opinions, letters, protocol trials, and editorials were considered not acceptable. Animals and in vitro studies were not eligible. All resulting titles were screened by hand, and suitable abstracts were accessed. If the abstract matched the topic and inclusion criteria, the full text was retrieved. If the abstract was not available and the title was out of topic, full-text retrieving was not performed. A cross reference of the bibliography of the full text was also conducted to identify additional studies. Full texts were saved in a dedicated folder shared between the authors in a private cloud.

The following data were extracted: first author and year of publication, paper title, study design, sample size, gender, age, statin(s) used, statin treatment duration, shoulder disease(s), and results. Data were extracted in Microsoft Office Excel version 16.89 (Microsoft Corporation, Redmond, WA, USA).

Moreover, we report here an original case that aids in the comprehension of statin-induced RCD as an illustrative example.

### Risk of Bias

The risk of bias (RoB) for the observational studies included in this review was assessed using the Newcastle–Ottawa scale (NOS) [15]. The NOS is a widely recognized and validated tool for evaluating the methodological quality of non-randomized studies, particularly cohort and case–control designs. It assesses three main domains: the selection of study groups, the comparability of groups, and the ascertainment of either the exposure or outcome of interest. The use of the NOS was chosen due to its established reliability and its frequent application in systematic reviews of observational studies, allowing for a standardized and transparent evaluation of study quality. The scoring also enables the integration of risk of bias as a potential moderator in further analyses. For this review, the assessment was performed independently by two authors (M.N. and V.G.), with disagreements resolved by consulting a third reviewer (P.C.). The results of the RoB are presented in Table 1.

## 3. Results

**Figure 1:**
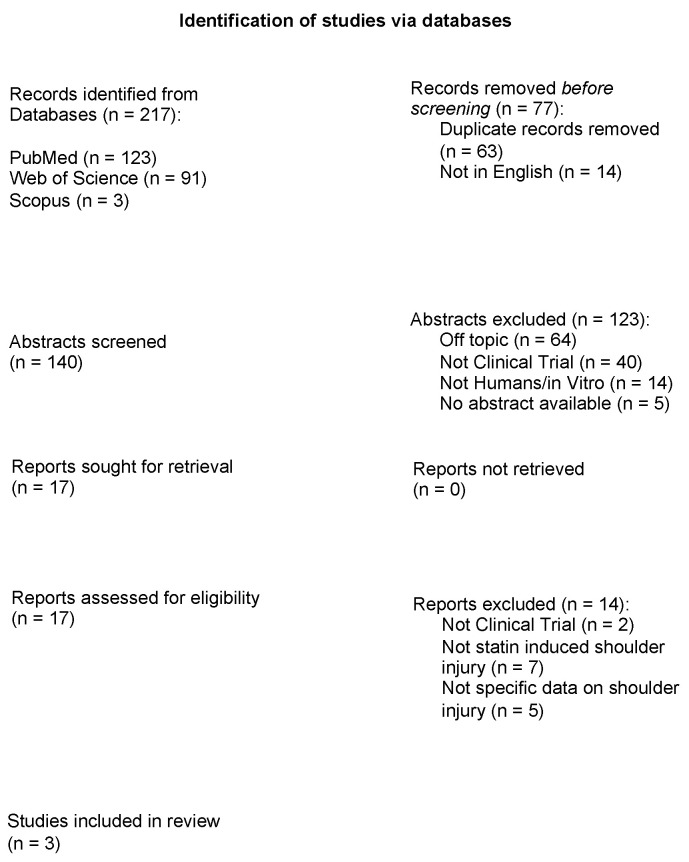
Study flow chart.

The comprehensive literature search identified 217 relevant studies. After eliminating duplicates (63) and papers not in English (14), 140 abstracts were screened for eligibility. Of these, 123 were excluded due to various reasons, including being off topic (64), non-clinical trials (40), not involving human subjects (14), or lacking available abstracts (5). Subsequently, the full texts of 17 remaining studies were retrieved. Fourteen of these were excluded for not being clinical trials (two), not focusing on statin-induced shoulder injury (seven), or lacking specific data on shoulder injury (five). Ultimately, three studies met the inclusion criteria and were included in this review (Figure 1, Table 2).

All the three included papers were cohort studies, involving a total of 843.917 subjects with various statin-induced tendinopathies. Among them 39.328 (5%) reported patients with statin-induced shoulder tendinopathy. The most commonly prescribed statins associated with this condition were atorvastatin and simvastatin, followed by rosuvastatin, pitavastatin, pravastatin, lovastatin, and fluvastatin. A lack of data about statin treatment duration was evident as only one paper reported this information [16]. Of the studies included in this review, only one explicitly specified RCD as the type of shoulder injury [16]. The remaining studies lacked clarity regarding the specific type of shoulder tendinopathy being investigated [17,18].

The findings from the three included investigations are conflicting. A large population-based cohort study by Kwak et al. [17] investigated the association between statin use and the development of shoulder tendinopathy. This study included 252.306 individuals, of whom 84.102 were statin users. The authors reported a significantly higher rate of shoulder tendinopathy in statin users compared to non-users. Atorvastatin and simvastatin were identified as the statins most frequently associated with this adverse effect. In contrast to these findings, Lin et al. [16] analyzed 498.678 patients and suggested that statin use might have a protective effect on shoulder health. Patients using statins exhibited a significantly lower risk of developing RCD compared to non-users. Lastly, Eliasson et al. [18] further supported the notion that statin use might increase the risk of tendinopathies. Their study, involving 92.933 participants, found that statin users had a higher incidence of shoulder tendinopathy compared to non-users.

### Case Presentation

A 49-year-old male was hospitalized for an acute coronary syndrome (ACS) and treated with percutaneous transluminal coronary angioplasty. Clinical history: semi-sedentary work, previous right shoulder pain as a result of past overuse injury related to sport activities, a family history of cardiovascular disease, smoking (>40 cigarettes/day), no high blood pressure, and no hypercholesterolemia. At the acute event time, the lipid profile was as follows: a total cholesterol of 153 mg/dL, an LDL cholesterol of 101 mg/dL, and an HDL of cholesterol 29 mg/dL. At discharge, clinicians prescribed atorvastatin, 20 mg/day. Three months after ACS, his creatine phosphokinase (CPK) level was in the range of normality. Then, clinicians increased the dose of atorvastatin up to 40 mg/day. After only few days since this increase, the patient developed symptoms on both shoulders, with pain and a reduction in range of motion (ROM). For this reason, he underwent an ultrasonography (US) of both shoulders that showed bilateral inflammatory RCD involving the infraspinatus, supraspinatus, and subscapularis. A further dosage of CPK showed a value of 207 U/L, which was outside the normal range, so the physicians decided to decrease the dosage of atorvastatin to 20 mg/day. After this reduction, there was a progressive resolution of the symptoms on the left shoulder up to a complete recovery of ROM with only occasional pain persistence. However, pain and functional limitation persisted in the right shoulder, which is the reason why the patient came to our attention, 15 months after the ACS.

**Figure 2:**
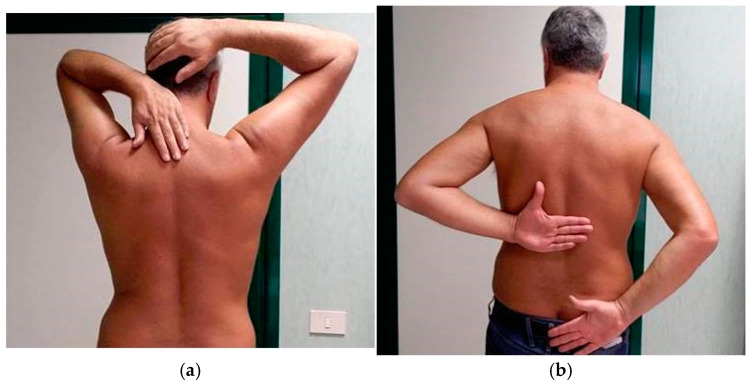
(**a**) Clinical evaluation of shoulders at T0. ROM limitation of the right shoulder in external rotation. (**b**) Clinical evaluation of shoulders at T0. ROM limitation of the right shoulder in internal rotation.

At our first examination (T0), the patient was very sore. As far as we are aware of, apart from the reported atorvastatin reduction, no other specific therapy was carried out in the period before our evaluation. We evaluated the shoulder ROM bilaterally and observed a painful limitation of the right shoulder in all planes, especially in internal rotation (Figure 2a,b). X-ray images of both shoulders showed only initial signs of osteoarthritis in the right acromioclavicular joint; no calcification was observed bilaterally.

We also administered evaluation scales to assess right shoulder function and pain: disabilities of the arm, shoulder, and hand (DASH) outcome measure [19], numerical rating scale (NRS) [20], short-form McGill pain questionnaire (SF-MPQ) [21] and Constant–Murley scale (CMS) [22]. We therefore planned a multimodal personalized IRP (Table 3) consisting of intra-articular injections of steroid and of high- and low-molecular-weight hyaluronic acid (HA), mesotherapy (intradermal injections), and therapeutic exercise, supported by existing validation of these therapeutic programs in the literature as well as in our clinical experience [23,24,25,26,27].

At T0, we started with the first intra-articular injection of a depot steroid (methylprednisolone acetate, 40 mg). The needle was directed into the acromiohumeral space through a posterior access. A second injection was performed after 7 days.

A new assessment was carried out 14 days after T0. The patient showed a reduction in pain and an improvement in ROM, so we prescribed therapeutic exercise with 45 min sessions three times a week for 4 weeks, and the exercise was carried out by a physiotherapist under the supervision of a physiatrist, including (a) the elevation of arm in the plane of scapula; (b) the early restoration of passive ROM; (c) a secondary stimulation of a programmed muscle contraction, initially isometric; (d) active counter-resistance work aimed at the recovery of the full ROM; (e) decoaptative reeducation through passive, active–static and active–dynamic recentering; and (f) proprioceptive training. On the same day, we performed the first of 5 weekly sessions (7 days apart) of mesotherapy, with multiple intradermal injections of betamethasone (1.5 mg/2 mL), 1 mL of lidocaine hydrochloride 2%, and 2 mL of a saline solution in the trapezius and deltoid regions. Corticosteroid use in mesotherapy has been reported in the literature, as described by Faetani et al. in their recent systematic review [28] and by a previous trial by Costantino et al. [29]. Betamethasone was chosen for its prolonged duration of action compared to other corticosteroids and its effective application in reducing inflammation and pain RCD [30,31,32].

At 21 day, the first intra-articular injection of high- and low-molecular-weight HA (32 mg + 32 mg/2 mL) was performed. A second one followed at a 7-day interval.

We comprehensively evaluated the patient before the last HA injection, which was 28 days after the beginning of treatment (T1), observing further improvement in internal rotation and pain reduction.

The treatment protocol was well tolerated, with an optimal adherence of the patient and without side effects.

Following the completion of the combined injection and therapeutic exercise program, the patient was educated on performing maintenance exercises independently. A follow-up schedule was established, with follow-up visits planned at three (T2) and six months (T3) after the initial evaluation.

Table 4 shows the scores of the evaluation scales that we used at the first observation (T0), before the last HA injection at 1 month (T1), and at the three-month (T2) and six-month (T3) follow-up.

**Figure 3:**
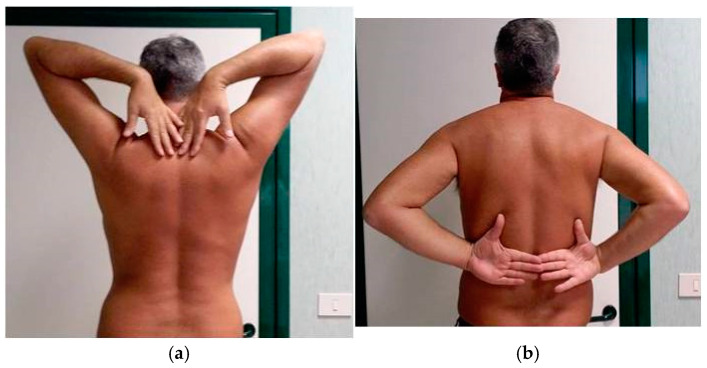
(**a**) Clinical evaluation of shoulders at T3, which is six months after the first observation. Complete recovery of external rotation of the right shoulder without pain. (**b**) Clinical evaluation of shoulders at T3, which is six months after the first observation. Complete recovery of internal rotation of the right shoulder without pain.

At T3, the painful symptomatology completely disappeared, and the ROM of the shoulder was markedly improved, with a slight limitation of internal rotation unperceived by the patient (Figure 3a,b).

A follow-up US examination revealed RCD signs of improvement. Then, the patient failed to attend subsequent follow-up appointments. When contacted by telephone one year after the initial evaluation, the patient reported continuing to take the reduced-dose statin. He reported complete remission of symptoms and expressed satisfaction with our IRP.

## 4. Discussion

Shoulder tendinopathy, particularly RCD, is a complex condition that necessitates a multidisciplinary approach for accurate assessment and effective personalized treatment [33]. Tendon injuries account for approximately 2.1% of reported side effects associated with statin therapy. A significant proportion of these cases, roughly 59%, occur within the first year of treatment [34]. The Achilles tendon, rotator cuff tendons, and bicep tendons are most commonly affected, and the precise molecular mechanism underlying the effects of statins on tendon cells remains to be fully elucidated [35]. In vitro studies have demonstrated that statin treatment can reduce extracellular matrix strength without significantly affecting total collagen levels. This suggests that alterations in the balance of matrix metalloproteinases may contribute to the observed changes in tendon tissue, while other reports showed decreased tenocyte migration and altered protein expression profiles [36,37,38]. In addition to the potential effects on the extracellular matrix, statins may also contribute to tendon injury through their intended mechanism of action: cholesterol synthesis inhibition. This could lead to compromised cell membrane integrity in tenocytes, potentially increasing their susceptibility to damage [39]. However, despite the growing body of evidence linking statins to an increased risk of tendinopathy, reports are conflicting. Animal and human studies have suggested that statins may exacerbate tendon degeneration [40,41,42,43,44], while others have reported beneficial effects on tendon healing [45,46,47,48].

Thus, our goal was to offer another perspective on the possible link between statins and tendinopathy, focusing on the tendons of the shoulder. The results of our review are in agreement with the conflict still present between beneficial [16] and harmful effects [17,36], and the case report shows a patient with RCD that may be related to statin intake. As in the study by Marie et al., in our case report, the association with atorvastatin was hypothesized because of the temporal relationship between the onset of symptoms and the increase in atorvastatin dosage, as well as the improvement in symptoms following a reduction in medication [34]. In alignment with previous research, our case report supports the association between atorvastatin and shoulder tendinopathy, corroborating findings from our review [17,36] and other reports [43,49]. While our findings contrast with those by Lin et al., it is important to note that their study did not specifically mention atorvastatin, which is one of the statin that has been more commonly linked to shoulder tendinopathy [16]. The patient also reported a previous injury in the right shoulder, which may explain the persistence of the symptoms only on this side, long after atorvastatin dose reduction. Notably, atorvastatin reduction by the cardiologist occurred not because of the shoulder symptoms but because the CPK value was elevated.

Considering RCD complexity, a personalized IRP incorporating steroid and hyaluronic acid injections, mesotherapy, and therapeutic exercise should offer a multi-faceted approach to managing RCD [50,51,52]. Steroid injections can provide temporary pain relief and reduce inflammation, facilitating subsequent rehabilitation efforts [53]. Hyaluronic acid injections, a viscosupplementation form of therapy, can restore synovial fluid viscosity and potentially promote tissue healing. Studies have demonstrated HA’s ability to reduce oxidative stress and apoptosis in human tenocytes, stimulate type I collagen expression, and modulate cell proliferation and the expression of procollagens and cytokines in shoulder synovial/capsular fibroblasts, thereby preventing adhesion formation and improving joint mobility [54]. Mesotherapy, a technique involving the injection of a customized mixture of substances, can improve local blood circulation and reduce pain [55]. Therapeutic exercise tailored to the individual patient’s specific needs and limitations is essential for strengthening the rotator cuff muscles, restoring the range of motion, and improving overall shoulder function [56,57,58,59]. By targeting both the underlying pathology and functional limitations, this comprehensive approach offered a widespread treatment for our patient. This led to improved symptoms, reduced pain, enhanced quality of life, and potentially avoided surgical interventions. Additionally, evaluation scale scores demonstrated a marked improvement, further supporting the effectiveness of the treatment.

### Limitations

The presented systematic review and original case report provide additional evidence supporting the potential link between statin use and tendinopathy, particularly RCD. However, it is important to note that a case report has well-known intrinsic limitations, including the potential influence of pre-existing factors. Further research is necessary to elucidate the precise mechanisms underlying the effects of statins on tendon tissue and to establish a definitive link between statin use and shoulder tendinopathy. Our review comes with limitations as well. The three included studies demonstrated a medium overall RoB. As a result, while the review could be considered robust, its findings should be interpreted with caution due to methodological limitations in the included studies. Moreover, while a meta-analysis is typically employed in studies similar to ours, in this case, such an exploration was not carried out because the included studies were heterogenous in participants numbers, drugs employed, and treatment duration. Lastly, when assessing the papers for quality of life or rehabilitation outcomes, nothing was found about these topics. We consider this as a limitation, given that otherwise we could have drawn meaningful connection between the observational data and the individualized approach described in the case report.

While the evidence remains inconclusive, clinicians should be vigilant in monitoring patients for signs of tendinopathy, especially those on high-dose statins or with a history of tendon injuries.

## 5. Conclusions

This study highlights the complex relationship between statin use and shoulder tendinopathy. While the evidence is evolving, it is crucial to consider the potential risks and benefits of statin therapy, especially for patients with a history of shoulder problems or those at a high risk for cardiovascular disease. Despite the undeniable protective role of statins in the primary and secondary prevention of cerebral and cardiovascular events, the possible side effects highlighted in musculoskeletal structures should not be neglected. A close follow-up of the patients is mandatory not only for monitoring cholesterol levels but also for monitoring side effects and consequently reducing statin dosage and starting an appropriate IRP. The efficacy of our multimodal personalized IRP could act as a cue for further studies aimed to outline guidelines for the management of statin-induced tendinopathies.

## Figures and Tables

**Table 1:** Risk of bias for observational studies (Newcastle–Ottawa Quality assessment scale).

Author, Year (Ref.)	Representativeness of the Study Population	Ascertainment of Exposure	Comparability of Cohorts on the Basis of the Design or Analysis	Assessment of Outcome	Follow-Up Long Enough for Outcomes to Occur	Adequacy of Follow up of Cohorts	Overall RoB
**Lin, 2015** **[16]**	Low	Medium	Medium	Medium	Unclear	Unclear	Medium
**Kwak, 2023** **[17]**	Low	Medium	Medium	Medium	Unclear	Unclear	Medium
**Eliasson, 2019** **[18]**	Low	Medium	Medium	Medium	High	Unclear	Medium

**Table 2:** Data from studies included in the review.

Authors, Year	Title	Study Design	Sample Size	Sex	Age: Mean, (Range)	Statin(s)	Statin Treatment Duration	Shoulder Disease	Results
Donghee Kwak et al., 2023 [17]	Effects of Statin Treatment on the Development of Tendinopathy: A Nationwide Population- Based Cohort Study	Cohort	Total: 252,306 Statin-induced shoulder tendinopathy: 10,605	Statin Users: F = 38,358 Non-users: F = 73,612	Statin Users: 49.78 ± 12.89 Non-users: 50.14 ± 14.11	Atorvastatin: 44.49% Simvastatin: 27.82% Rosuvastatin: 12.03% Pitavastatin, pravastatin, lovastatin, and fluvastatin: 15.67%	/	Shoulder tendinopathy (generic)	Statin users: higher rates of tendinopathy development than non-users regardless of statin type and cumulative dosage. Atorvastatin and simvastatin users: greater rates of tendinopathy development across all types of tendinopathy compared. Rosuvastatin users: trigger finger, elbow epicondylitis, and shoulder tendinopathy showed higher rates.
Tony Tung-Liang Lin et al., 2015 [16]	The Effect of Diabetes, Hyperlipidemia, and Statins on the Development of Rotator Cuff Disease	Cohort	Total: 498,678 Statin-induced Rotator Cuff Disease: 26,664	F = 245,277	48.8 ± 14.0	Rosuvastatin, simvastatin, and others (not specified)	>28 days	Rotator Cuff Disease	Statin use associated with a lower risk of developing Rotator Cuff Disease when compared with non-statin use
Pernilla Eliasson et al., 2019 [18]	Statin treatment increases the clinical risk of tendinopathy through matrix metalloproteinase release—a cohort study design combined with an experimental study	Cohort	Total cohort: 92,933 Statin-induced Shoulder tendinopathy: 2059	F = 1102	Four groups: 68.4 ± 7.8–69.5 ± 9.8–69.5 ± 8.5–66.6 ± 9.7	Simvastatin (69%), atorvastatin (24%), rosuvastatin (4%), and pravastatin (2%)	/	Shoulder tendinopathy (generic)	Higher incidence of shoulder tendinopathy in patients who were current users of statins compared to non-users

**Table 3:** Multimodal individual rehabilitation project.

T0	First Assessment: Comprehensive Evaluation Intra-Articular Injection of Steroid
**day 7**	intra-articular injection of steroid
**day 14**	mesotherapy start of therapeutic exercise
**day 21**	intra-articular injection of HA mesotherapy therapeutic exercise in progress
**T1—day 28**	comprehensive evaluation intra-articular injection of HA mesotherapy therapeutic exercise in progress
**day 35**	mesotherapy therapeutic exercise in progress
**day 42**	mesotherapy end of therapeutic exercise

**Table 4:** Scores of the evaluation scales at T0, T1, T2, and T3.

	DASH	NRS	SF-MPQ	CMS
**T0**	69.2/100	7/10	46/60	38%
**T1**	23.3/100	2/10	16/60	81%
**T2**	4.2/100	0/10	0/60	93%
**T3**	0.8/100	0/10	0/60	98%

## Data Availability

The original contributions presented in this study are included in the article. Further inquiries can be directed to the corresponding author.
